# Characteristics of the stalking campaign: Consequences and coping strategies for men and women that report their victimization to police

**DOI:** 10.1371/journal.pone.0229830

**Published:** 2020-02-28

**Authors:** Daniela Acquadro Maran, Antonella Varetto, Ilenia Corona, Maurizio Tirassa

**Affiliations:** 1 Department of Psychology, Università di Torino, Torino, Italy; 2 Città della Salute e della Scienza—Corso Bramante, Torino, Italy; University of Sao Paulo Medical School, BRAZIL

## Abstract

The study analysed cases reported to police by men and women who were victims of stalking. The objective was to describe the characteristics of the stalking campaigns experienced by men and women, their consequences, and the coping strategies adopted by the victims, as they are recorded in police case files. All the information was collected in three cities in the Northwest of Italy. Analyses were performed on 271 files classified by police officers as cases of stalking, reported by men (87, 32.1%) and women (184, 67.9%). The study revealed that men tended to let the stalking campaign last for a longer time than women before turning to the police. Procrastination had some consequences, especially in the emotional sphere, that affected the victim’s wellbeing. Moreover, the coping strategies used by men victims were not effective and even risked to hamper the work of the police officers intervening and investigating on the case.

## Introduction

Stalking has been a focus of interest of social scientists since the 1990's. In Italy, where the study we present here was conducted, it has been considered a crime since 2009. Stalking is commonly defined as a set of repetitive, unwelcome, and intrusive behaviours directed towards an individual who consequently experiences apprehension, annoyance, and/or fear for her/his safety or the safety of others [1]. According to Tjaden and Thoennes [2, p. 1], ‘stalking generally refers to harassing or threatening behaviour that an individual engages in repeatedly, such as following a person, appearing at a person’s home or place of business, making harassing phone calls, leaving written messages or objects, or vandalizing a person’s property. The same authors conducted one of the first investigations in the US involving the general population (2000). In a sample of 16,000 people (50% men), 0.5% of men and 4.1% of women reported to having been victims of stalking in the previous 12 months. 27.8% of women victims, but less than half as many men (13.4%) turned to the police. Similar figures were found by Hall [3]. In her investigation, most of the men victims had been stalked by a woman; they did not turn to the police for fear of not being believed or taken seriously. An investigation conducted by Walby and Allen [4] in Great Britain found that 8.9% of 11,028 men surveyed had been victims of stalking. The perpetrator was an acquaintance in 70% of the cases, an intimate in 8%, and unknown in the others. A meta-analysis by Spitzberg, Cupach and Ciceraro [5] found that, on average, 14% of men were victims of stalking over the course of their lives. The figure for women was 29%. An explanation for the lower percentage of male victims could be a lower tendency of men to identify themselves in the role of the victim, which would in turn make them less likely to seek help [6]. Bjerregaard [7]surveyed 788 students of a U.S. university. 29 men (10.9% of the relevant sample) and 122 women (24.7%) said that they had been subjected to stalking. These figures are in line with those reported above. The average stalking campaign against a man lasted more than double than against a woman (more than 26 weeks and, respectively, approximately 12 weeks). Purcell, Pathé and Mullen [8]hypothesize that this might be due to the stalker of men more often being a woman than another man: indeed, the stalking campaigns conducted by women generally last longer than those conducted by men (*Me*_women_ = 22 months, range = 2–240 months; *Me*_man_ = 12 months, range = 1–240 months). Spitzberg, Cupach and Ciceraro's [5] meta-analysis also found that men reported being pursued longer than women, with a difference of almost 5.5 months. Differences between the experiences of victimized men and women were also observed in terms of the frequency of contacts from the perpetrator. Frequency of contacts has been associated with a greater likelihood of serious violence [9,10]; men generally reported fewer contacts than women [5].

As mentioned above, men are more often stalked by a woman than by other men, confirming that the phenomenon has a mainly intergender nature [11]. According to Purcell, Pathé and Mullen [8], the most frequent motive behind a stalking campaign is the desire to establish or re-establish a relationship. Moreover, Ferreira and Matos [12] found that the majority of victims of stalking where the perpetrator was an ex-partner had reported partner abuse during the relationship.

## Behaviours characterizing the stalking campaign

McEwan and colleagues [13] and Spitzberg and Cupach [14] propose to categorize the behaviours that typically characterize a stalking campaign as:

- unwanted communication (by any means of contact, e.g., telephone calls, e-mails, letters or cards, text messages, and so on),
- unwanted approaches (e.g., following; visits to or waits outside the home or the workplace, and so on),
- harassment and intimidation (threats to the victim, his/her children, partner or other family member; seeking information about the victim from third parties; defamation, and so on).

Macrì and colleagues [15] found that the relative frequency of the various stalking behaviours was different when the victim was a man or a woman: unwanted communication (such as telephone calls or text messages) and acts of vandalism were comparatively more frequent toward men, while unwanted approaches (such as loitering or following) or harassment and intimidation (e.g., threats) were comparatively more frequent toward women. This might be due at least in part to the perpetrator's fear of reaction or payback on the part of the victim. Sheridan, Gillett and Davies [16] found that men are less prone than women to consider certain behaviours as stalking. They asked a sample of 95 victims of stalking to pick from a list of 42 possible behaviours those to which they had been subjected over the course of their lives. Men (7% of the sample) picked a total of 10 such behaviours, while women (92%) picked 32. A possible explanation of this difference might be that men–as discussed above—are less inclined to view themselves as victims, perhaps because of a lower tendency to consider these behaviours as particularly serious [16].

### Consequences of the stalking campaign

Littel [17] described stalking as ‘soul destroying’; indeed, it does have physical and emotional consequences for the victims. Direct physical consequences may include injuries inflicted by the stalkers which may or may not require medical care [18]. Indirect physical consequences may include stomach trouble, weight fluctuations, headaches, weakness, and sleep disorders [19]. Emotional consequences may include anger, anxiety, depression, fear, paranoia, confusion, distrust, and suicide ideation [14, 20]. Of course the victim's social life, capability to work, economic conditions, life decisions etc. may also suffer during the stalking campaign and its aftermath.

In their investigation of health care professionals who were victims of stalking, Acquadro Maran and Varetto [21] found no differences between women and men as for the consequences, except that anger and minimal anxiety (both state and trait) affected the latter more than the former. Regarding fear specifically, Owen [22] found that women victims of stalking are much more fearful than men; however, Sheridan and Lyndon [23] found that women have higher levels of fear of crime than men in general anyway, including a specific fear of stalking.

### Coping strategies

Victims may and do use a variety of strategies to cope with stalking. Spitzberg, Nicastro and Cousins [24]found that the main difference between men and women victims was that men were less prone to ask for help. Spitzberg and Cupach [25] proposed a categorization of the strategies: *moving inward* (e.g., seeking a therapist, using drugs), *moving outward* (e.g., seeking social support), *moving towards* (e.g., reasoning with the stalker), *moving against* (threatening or harming the stalker), and *moving again* (attempting to escape the stalker). Reconsidering Spitzberg and colleagues’ [24]work in retrospect, men turned out to have a lesser tendency than women to move outward. In Amar's [26] study, the most frequent strategies employed by women victims were to change their routines, to just ignore the stalker or to confront him/her.

### Current study

Stalking is a crime in Italy. Act 38/2009 punishes “whoever, with repeated conduct, threatens or harasses someone in such a way as to cause a lasting and serious state of anxiety or fear or to generate a well-founded fear for one's own safety or that of a next relative or that of someone to whom one is linked by an affective relationship, or to force him or her to alter his or her own life habits” (art. 612 (*b*)) [27]. According to data published by the Italian Ministry of Justice [28], out of approximately 51,000 cases of stalking reported to the police from 2009 to 2014, more than 11,300 (22.3%) concerned male victims.

The protocol for dealing with this crime provides that, when an alleged victim turns to the police, the officer asks to present materials and/or testimonies in support of the complaint. If, in the opinion of the agent, there is grounds for this, a report is filled in. The alleged stalker is then summoned separately and confronted with the materials presented by the alleged victim. If he or she is unable to offer alternative explanations, he or she is warned of the seriousness of the conduct and the judicial risks he or she will face in the case of relapse. However, if the behaviours reported have a different, non-stalking explanation he or she may sue the self-styled victim for slander. After this warning, stalking behaviours cease in 59.8% of the cases and decrease in 16.6% [29]. Those who persist are prosecutable in court. The study we describe here aimed to analyse the cases reported to police by men and women who were victims of stalking. To examine the police reports makes sense because, as discussed above, (*a*) they are only filled in when the police officer believes that there is sufficient supporting evidence to do so, and (*b*) a large majority of the cases of stalking are resolved in this phase, while only a small fraction gives rise to a real criminal proceeding: even the latter, however, will almost always have gone through this first handling stage. Police records have already been used for research on the victims of stalking and other violent crimes (e.g., [30,31]). The goal here was to describe the characteristics of the stalking campaign experienced by men and women, their consequences, and the coping strategies adopted by the victims, as recorded in police case files. All the cases reported had taken place in three cities in the northwest of Italy. Based on Italian standards, of the three municipalities, one is considered a large city and the other two are considered smaller cities or towns.

This study aims to understand the differences between men and women in the experiencing of being a victim of stalking: particularly in men, this could be useful to identify barriers to help-seeking as well as provide insight to police officers [32]. The hypotheses of the study were the following:

- We expected the characteristics of stalking reported to police by men to be different from that reported by women, namely, in the former case: we expected there to be a prevalence of female stalkers (H1a); we expected the duration of the stalking campaign to be longer (H1b); we expected the frequency of direct contacts to be lower (H1c); we expected the motives to be mostly related to establishing or re-establishing a relationship (H1d).
- In regard to the types of stalking behaviours involved, we expected that men victims would experience more unwanted communication and acts of vandalism than women and less unwanted approaches, harassment and intimidation (H2a); we also expected that they would experience a lower variety of behaviours (H2b).
- We expected men and women to experience the same kinds of psychological consequences (H3a), except for fear, which we expected to be less frequent in men (H3b).
- In regard to coping strategies, we expected men would be less prone to ask for help (H4a).

## Methods

### Participants

The data were collected with the cooperation of four distinct police units. These belonged to two different types of forces, the Polizia di Stato (State Police) of all three cities, and the Polizia Municipale (Municipal Police) of the larger one. Among the responsibilities of the State Police of course is to protect the citizens from violent behaviours (see http://www.poliziadistato.it); of course this role of theirs is common knowledge of the population. The Municipal Police were chosen because, differently to the other forces, they have set up a unit specializing in cases of stalking. This is well known locally because they offer information days open to the public to spread awareness about stalking, organize training courses in schools, and give out information brochures.

Analyses were performed on 271 files classified by police officers as cases of stalking (based on art. 612*/bis* of the Italian criminal law mentioned above). All the files contained sociodemographic details about the victim (e.g., sex and age), details about the stalker (e.g., sex, if known), a description of the relationship between the victim and the stalker, and a description of the stalking campaign (duration, frequency of contact, behaviours enacted by the presumed offender, consequences, and coping strategies on the part of the victim).

Of the 271 victims, 87 (32.1%) were men, 184 (67.9%) women. Overall, victims were on average 40.71 years old (range, 12–92; *s*.*d*. = 13.63). The majority were single (89, 32.8%), 42 (15.5%) were divorced, 40 (14.8%) were married, 14 (5.2%) were engaged, 11 (4.1%) cohabited, and one (0.4%) was a widow. Information about marital status was not available in other 74 cases.

Most perpetrators were males (203, 74.9% vs. 66 females, 24.4%). This information was unreported in two cases. The stalker was an ex-partner in 170 cases (62.7%) and an acquaintance in 90 cases (33.2%); in 11 cases (4.1%), his or her identity remained unknown. The stalkers were, on average, 43.24 years old (range, 12–82; *s*.*d*. = 13.24); age was undetermined in 17 cases. The stalker’s marital status was single in 101 cases (37.3%), divorced in 47 (17.3%), married in 16 (5.9%), engaged in three (1.1%), cohabitant in two (0.7%), widowed in one (0.4%), and undefined in the remaining 101. 32 perpetrators stalked more than a person (for example the ex-partner and his/her parents). The average duration of the stalking campaign at the time of the report was 96 weeks (range, 1–1060 weeks; *s*.*d*. = 159), that is 1.8 years.

### Measure

The police reports of stalking offences available from 2009 (when, as mentioned above, relevant laws were introduced in Italy) to 2018 were transcribed. In compliance with the Italian law on personal data protection, all sensitive data (e.g., name/surname and identification codes of the persons involved) were stripped. The remaining, non-sensitive data were then analysed with a grid obtained from a modified version of the questionnaire on stalking constructed by the Network for Surviving Stalking (NSS) and Dr. Lorraine Sheridan (Forensic Psychologist, Curtin University, Australia). This questionnaire has already been used in research on stalking in the Italian context [33]. It covers the following information, when available:

- the demographic details of the victim: sex, age, marital state (3 items);
- the demographic details of the stalker: sex, age, marital state (3 items);
- the duration of the stalking campaign (1 item);
- the frequency of behaviours, categorized as: one or more times a day, one or more times a week, one or more times a month, or less than one time a month (1 item);
- the nature of the relationship between the victim and the stalker, categorized as ex-partners, acquaintances or unknown (1 item each, yes/no response);
- the motives of the campaign as reported by the victims, e.g., ‘quarrels with neighbours’ (5 items, yes/no responses)
- third parties possibly harassed in their turn during the campaign, e.g., ‘son/daughter’ (7 items, yes/no responses);
- the presence of domestic violence, with possible responses being no; yes, physical violence; yes, emotional violence; or yes, physical and emotional violence (1 item each, yes/no responses);
- the kinds of stalking behaviours, e.g., ‘the stalker sends e-mails to the victim’ (18 items; yes/no responses);
- the consequences of the stalking campaign in terms of physical (7 items, e.g. headache) and emotional (12 items, e.g. fear) symptoms; possible responses also included ‘other symptoms’;
- lastly, the coping strategies adopted, e.g., ‘talk to stalker’ (13 items; yes/no responses).

### Procedures

The data were collected by one of the authors of this paper with help from research assistants trained by the same researcher. One of the authors contacted the four police units, which all agreed to participate in the project. During a formal meeting with the chiefs of the police forces and their delegates, the aims and procedure of the research and its implications were explained and discussed. After the final approval of the project, all the officers were informed that their units were going to be involved in an investigation of the stalking reports. One officer from each unit was appointed to supervise that the proceedings would comply the privacy requirements of the Italian law (namely, redaction of sensitive data). The remaining contents of the reports were transcribed into a new SPSS file. Data collection took about one week for unit. The resulting file was processed with SPSS 22 to produce mainly descriptive and inferential statistics. Descriptive measures (frequencies, means ± SD) were calculated for all test variables; χ^2^ tests were used to measure the differences. T-tests were used to examine the differences between the mean scores of the sexes for each variable; the results were considered statistically significant for *p* < .05. Correlations were calculated between the duration of the stalking behaviour, the type of stalking behaviours, the type of physical and emotional symptoms reported, and the coping strategies used by men and women as listed in the questionnaire.

### Ethical statement

This research conformed to the provisions of the Declaration of Helsinki in 1995, as revised in the Edinburgh meeting of 2000 [34]. All the relevant ethical guidelines were followed, including compliance with the requirements of Italian legislation.

More specifically: (*i*) *Approval of the research project*: The research project was approved by the Ethics Committee of the Università di Torino before the study began (n. 23622-15/07/2015). Since there was no medical treatment or other procedures that could cause biological, psychological or social harm to the police officers involved, additional ethical approval was not required. (*ii*) *Police personnel*: The chief of each police unit involved appointed one of their officers to collaborate to the research. These delegates were authorized to log into the files of their unit; their collaboration consisted in general help to the researchers as well as, specifically, in redacting all the sensitive data contained in the reports. The letter of informed consent given to both the chiefs and the delegates clearly stated the goals of the research, the voluntary nature of participation, the types of data investigated and their statistical processing, and the anonymity of the procedures employed. (*iii*) *Denouncers*, *i*.*e*., *presumed victims*: The oral informed consent of whomever asks to file a report to the police is routinely obtained by the officers during the interview. This is also the case with reports of stalking. The report and all the information it contains is then undersigned by the denouncer. By this act, he or she authorizes the police to treat the information gathered according to further investigative and judiciary needs and proceedings, as well as to anonymized elaboration for statistical and scientific purposes. It is important to notice here that we as researchers had no personal contact with the denouncers: we only had access to their reports as collected at previous times by the police officers. The officers involved took care to eliminate the personally identifiable information from a copy of the files before passing them to us. Nobody, including the officers, received any compensation for their participation.

## Results

### Characteristics of the stalking campaign

The 87 men that reported being stalked to the police were, on average, 42.15 years old (*s*.*d*. = 14.92; range, 12–82). The average age of the 184 women was 40.04 years (*s*.*d*. = 12.96; range, 14–92) (*t* = 1.87; *p* = n.s.). Stalkers were women in the majority of cases (n = 55, 63.2%) when the victim was a man. This percentage fell to 6% when the victim was a woman (two women victims did not answer this question—see Table 1). Male stalkers were, on average, 42.51 years old (s.d. = 14.03; range, 12–69); female stalkers were, on average, 43.55 years old (*s*.*d*. = 12.93, range 16–82) (*t* = -.57; *p* = n.s.). On average, stalking targeting men lasted 130.44 weeks before the victim turned to the police (*s*.*d*. = 222.32; range, 1–1060), with a frequency of contact of about once a week. Stalking targeting women lasted an average of 79.05 weeks (*s*.*d*. = 115.79; range, 1–1060) (*t* = 2.38; *p* = .018), with an average frequency of contact of one or more times a day (*p* < .05). Regarding the relationships between the stalkers and their victims, men were more often targeted by acquaintances than women were, but less often by ex-partners. When the stalker was a former partner, men reported having experienced physical or emotional violence during the relation less often than women. The most frequent motives reported for the stalking campaign were a quarrel with neighbours and an unknown reason when the victim was a man, and the end of a relationship when it was a woman. Other motives mentioned were quarrels at school (men = 3, 3.4%) or within the family (men = 2, 2.2%; women = 1, 0.5%), and racial intolerance (men = 3, 3.4%; women = 3, 1.6%).

**Table 1:** Characteristics of the relationship victims-stalker and motive for the beginning of the stalking campaign in men and women that report to police (N = 271).

	Men *n* = 87 *n* (%)	Women *n* = 184 *n* (%)	χ^2^	*p*
Gender: Male Female	32(36.8) 55(63.2)	171(92.9) 11(6)	103.92 103.92	.000 .000
Frequency: One or more time a day One time a week One or more time a month	35(40.2) 39(44.8) 3(3.4)	100(54.3) 56(30.4) 0(0)	4.71 5.38 6.42	.021 .015 .032
Relationship:				
Ex-partner	37(42.5)	133(72.3)	22.37	.000
Acquaintance	45(51.7)	45(24.5)	19.80	.000
Unknown	5(5.7)	6(3.3)	0.94	n.s.
Domestic Violence:				
Physical Violence	1(1.1)	0(0)	1.56	n.s.
Emotional Violence	3(3.4)	14(7.6)	3.04	n.s.
Physical and emotional violence	3(3.4)	46(25)	17.87	.000
Disagreements in workplace	8(9.2)	12(6.5)	0.62	n.s.
End of the relationship	37(42.5)	133(72.3)	22.37	.000
Establish a relationship	8(9.2)	19(10.3)	0.08	n.s.
Quarrels with neighbors	12(13.8)	3(1.6)	16.71	.000
Unknown	14(16.1)	13(7.1)	5.37	.020

*Note*. χ^2^ = chi-square; *p* = p-values; n.s. = not statistically significant

### Behaviours characterizing the stalking campaign

Whether targeting men or women, the stalking campaigns were typically characterized by a variety of behaviours (Table 2). On average, men experienced 5.7 (*s*.*d*. = 3.05) different behaviours, and women experienced 6 (*s*.*d*. = 3) (*t* = -.73, *p* = n.s.). In particular, men were subject to more acts of vandalism, searches for information, and spreading of lies than women; other behaviours included e.g. sending photos of the victims as postcards. Instances of following and threats were instead experienced less frequently by men than by women.

**Table 2:** Behavior characterizing the stalking campaign in men and women that report to police (N = 271).

	Men *n* = 87 *n* (%)	Women *n* = 184 *n* (%)	χ^2^	*p*
Acts of vandalism	23(26.4)	32(17.4)	3.53	.045
Asking for information	12(13.8)	12(6.5)	4.26	.036
Children’s harassment	10(11.5)	30(16.3)	0.81	n.s.
Cyberstalking	20(23)	31(16.8)	1.95	n.s.
Following	38(43.7)	117(63.6)	7.45	.005
Loitering	43(49.4)	106(57.6)	0.78	n.s.
Sending gift	8(9.2)	16(8.7)	0.06	n.s.
Sending e-mail, letters or cards	27(31)	47(22.8)	2.62	n.s.
Spreading lies	18(20.7)	8(4.3)	19.56	.000
Text message	36(41.4)	97(52.7)	2.24	n.s.
Telephone calls	47(54)	119(64.7)	1.86	n.s.
Threats	29(33.3)	124(67.4)	27.87	.000
Threats of children’s harassment	7(8)	14(7.6)	0.05	n.s.
Visiting home	32(36.8)	86(46.7)	1.75	n.s.
Visiting workplace/school	37(42.5)	62(33.7)	2.90	n.s.
Waiting outside home	29(33.3)	85(46.2)	2.72	n.s.
Waiting outside workplace/school	34(39.1)	74(40.2)	0.01	n.s.
Other	8(9.2)	2(1.1)	11.60	.002

Note. χ2 = chi-square; p = p-values; n.s. = not statistically significant

The duration of the campaigns against men correlated with specific types of stalking behaviors, namely the harassment of the victim’s children (*r* = 35; *p* = .006), visiting the victim’s home (*r* = 31; *p* = .019), and spreading lies against the victim or related persons (*r* = 27; *p* = .014). No such correlation was found with women victims.

### Consequence of the stalking campaign

Men who were victims of stalking suffered from as many consequences (Table 3) as women. On average, men experienced 0.6 physical symptoms *(s*.*d*. = 1) whilst women experienced 0.7 (*s*.*d*. = 0.71; *t* = 1.37, *p* = n.s.). Of the physical consequences, headaches, sleep disorders, and weakness were reported more often by men, whilst injuries were reported more often by women. Of the emotional consequences, men reported 2.6 emotional symptoms *(s*.*d*. = 1.86) whilst women reported 2.2 (*s*.*d*. = 0.74; *t* = 2.64, *p* = .009). Aggressiveness, anger, confusion, irritation, lack of confidence, and panic attacks were experienced more frequently by men than by women; apprehension and fear less frequently. Correlation analysis found that in men victims an increase in the variety of stalking behaviours was related to an increase in emotional consequences (*r* = .27, *p* = .018); in women victims, an increase in emotional consequences was related to an increase in coping strategies (*r* = .19, *p* = .009).

**Table 3:** Physical and Emotional symptoms in men and women reporting stalking case (N = 271).

	Men *n* = 87 *n* (%)	Women *n* = 184 *n* (%)	*χ*^*2*^	*p*
Physical symptoms:				
Headache	4(4.6)	0(0)	9.23	.008
Nausea	1(1.1)	2(1.1)	0.01	n.s.
Sleep disorder	26(29.9)	30(16.3)	8.42	.004
Stalker’s injuries	3(3.4)	32(17.4)	9.19	.001
Stomach trouble	2(2.3)	6(3.3)	0.12	n.s.
Weakness	17(19.5)	20(10.9)	4.79	.025
Weight change	4(4.6)	3(1.6)	2.39	n.s.
Emotional symptoms:				
Aggressiveness	4(4.6)	1(0.5)	5.78	.033
Agoraphobia	5(5.7)	4(2.2)	2.67	n.s.
Anger	11(12.6)	3(1.6)	15.80	.000
Apprehension	64(73.6)	168(91.3)	8.94	.003
Confusion	7(8)	2(1.1)	9.63	.004
Fear	65(74.7)	174(94.6)	16.09	.000
Irritation	15(17.2)	14(7.6)	6.67	.011
Lack of confidence	6(6.9)	1(0.5)	10.16	.004
Panic attacks	11(12.6)	5(2.7)	11.69	.001
Paranoia	6(6.9)	12(6.5)	0.07	n.s.
Sadness	8(9.2)	11(6)	1.22	n.s.
Suicidal thoughts	0(0)	1(0.5)	0.45	n.s.

*Note*. χ^2^ = chi-square; *p* = p-values; n.s. = not statistically significant

Physical and emotional consequences for both men and women worsened with the duration of the stalking campaign. For men victims, the duration of the campaign correlated with weakness (*r* = .39, *p* = .001), weight change (*r* = .25, *p* = .027), stomach trouble (*r* = .45, *p* = .000), and sleep disorders *(r* = .30, *p* = 008). For women victims, the duration correlated with being injured by the stalker (*r* = .26, *p* = .001) and weakness (*r* = .21, *p* = .007). Among the emotional consequences, the duration of the campaign correlated with fear (*r* = .34, *p* = .003) and irritation (*r* = .29, *p* = .010) in men victims, while no specific correlation was found for women.

### Coping strategies

Men victims used a variety of strategies to cope with the stalking campaign (Table 4). On average, men adopted 3.2 *(s*.*d*. = 2) strategies, women 2.4 (*s*.*d*. = 1.4; *t* = 3.7, *p* = .000). More in detail, men were more inclined than women to answer to and reason with the stalker, e.g., talking to him/her. They were, however, less inclined than women to collect evidence and devise a safety plan.

**Table 4:** Coping strategies in men and women reporting stalking case (N = 271).

	Men *n* = 87 *n* (%)	Women *n* = 184 *n* (%)	χ^2^	*p*
Answer to email	6(6.9)	3(1.6)	5.72	.026
Answer to telephone call	14(16.1)	7(3.8)	14.01	.000
Answer to text message	13(14.9)	6(3.3)	13.82	.000
Ask to stalker to stop	42(48.3)	18(9.8)	55.75	.000
Caught in the act	27(31)	23(12.5)	15.95	.000
Change daily routine	46(52.9)	128(69.6)	4.55	.024
Collect evidence	53(60.9)	154(83.7)	13.09	.001
Decrease social contact	8(9.2)	37(20.1)	4.18	.028
Drugs	6(6.9)	12(6.5)	0.07	n.s.
Increase social contact	4(4.6)	1(0.5)	5.87	.032
Safety plan	15(17.2)	44(23.9)	0.95	n.s.
Seek a psicologist	4(4.5)	10(5.4)	0.06	n.s.
Talk to stalker	29(33.3)	11(6)	39.03	.000

*Note*. χ^2^ = chi-square; *p* = p-values; n.s. = not statistically significant

The duration of the stalking campaign correlated with the adoption of specific coping strategies, namely having a safety plan (*r* = .38, *p* = 001) and asking the stalker to stop (*r* = .29, *p* = 009) for man victims, and turning to a psychologist (*r* = .48, *p* = 000) and to have a safety plan (*r* = .20, *p* = 011) for women victims.

## Discussion

The analysis of the cases reported to police showed that the characteristics of the stalking campaign, the kind of behaviours enacted by the stalker, the physical and emotional consequences of the campaign, and the coping strategies experienced by men and women were different. Considering the nature of the relationship between the victims and their stalkers, the findings confirmed that stalking is mostly–but, of course, not always–intergender [35]. Most men victims were stalked by a woman; thus, hypothesis H1a was confirmed. Considering the duration of the stalking campaign, in accordance with H1b, men let the stalking campaign last much longer than women before turning to the police, which also confirms data from Spitzberg, Cupach and Ciceraro’s [5]meta-analysis. Moreover, as expected, the frequency of the stalking behaviours was lower against men than against women, which confirmed H1c. Considering the motives of the campaign, in most cases men were not stalked with the aim to establish or re-establish a relationship; therefore, H1d was *not* confirmed. Men who reported to the police were stalked by an acquaintance more often than women. Consistently, the motives mentioned to the police were mostly related to quarrels with neighbours and others. This is very interesting and should be studied in more depth if a higher number of reports were available, and if the questionnaire used by the police investigated in more depth the motives of the stalking campaign and the reasons for reporting to police. Considering the behaviours that characterized the stalking campaign, men, as expected, were more frequently targeted with acts of vandalism than with threats. They also experienced more acts of intrusive communication and more instances of a third person also being targeted by the stalker. Thus, hypothesis H2a was only partially confirmed, which suggests that further investigation should explore the involvement of third parties like partners, spouses, relatives, friends, etc. as secondary victims [36]. This could in turn help better understand these victims' reasons for reporting to the police.

The duration of the stalking campaign for men before reporting to the police was related to behaviours targeting the private sphere, namely children, home, and reputation. Moreover, both men and women were subject to an array of abusive behaviours; thus, H2b was *not* confirmed. Being the target of a stalking campaign affects men and women in different ways. Men victims suffered from physical and emotional symptoms and panic attacks more frequently than women; thus, H3a was *not* confirmed. These data are very interesting insofar as they probably go to show that stalking against men is not easily considered in our culture: men just do not expect to be victims of stalking [37]. Stalking as a general phenomenon and its actual structure and dynamics are perceived in different ways by men than by women. For example, men victims tend to not perceive approaches as stalking behaviours [38]; they tend to expect to be targeted with direct violence [39], rather than repetitive, intrusive and elusive behaviours [16]. Maybe in part because of this, they experienced less fear than women; our expectation H3b was thus confirmed. To investigate the general public’s representation of stalking and its motives might help understand whether and to what extent the perception that the victims have of the campaign to which they have been subject is shaped by the representations of stalking in the media and in the public opinion. Also, the victims' reports might be directly useful to improve social communication campaigns about stalking. The analysis of how the victims cope with stalking showed that men tended to adopt a greater variety of strategies than women. In most cases, men used *moving outward* and *moving toward*, whilst women used *moving again* and *moving inward*. This confirmed hypothesis H4a. Consistently with the standard idea of masculine behaviours, the men victims' responses to the stalker were often direct, without a fear of confrontation that instead tends to characterize women victims [40]. To increase social contact is a defence mechanism involving a search for group connection and solidarity, but does not normally provide solutions to the problem of being stalked. To directly confront the stalker is ineffective in most cases [41,42]. To change one’s habits and routines and to collect evidence are better strategies insofar as they may help reporting the campaign to the police, because the former is directly included in the definition of the Italian laws about stalking, and the latter of course facilitates the task of the police [11,21].

### Limitations

This research has some limitations that should be mentioned. First, all the police units from which the files were collected were operating in North-western Italy; the sample may not have been entirely representative of the national population. The results should therefore be considered fully relevant only to the territory sampled. Second, the sexual orientation of the victims was unavailable in most reports; further research on a wider sample should address the police forces to investigate the issue and the characteristics of the campaigns targeted to specific gender orientations. This could also help understand the role of acquaintances and strangers when stalking is related to sexual orientation [43]. Third, we were only allowed by the police forces to log into their files when they were classified as stalking (art. 612/bis of the Italian criminal law): reports in which stalking was subsumed by other more serious crimes (e.g., attempted murder) were not available. Since the evolution of stalking toward graver offenses is more frequent when a woman is the victim [44], stalking against men could be overrepresented in our sample. Fourth, while the police force units employ a standard scheme to organize and record the statements provided by the victims, the actual dialogues between the officers and the victims may direct the emphasis toward specific features of the stalking campaign. Adopting a format with both open and closed questions might yield better descriptions on the part of the victims of the characteristics of the campaign, the stalker's behaviour, the consequences, and the coping strategies deployed [21]. Such format might also allow the police officers to gather all the relevant information about the case as well as to help the victims enact effective coping strategies should the campaign continue, e.g., seeking help from bystanders (see [45]). In the terms of this study, a better format of the police reports might also yield a better understanding of the differences between men and women (or different groups and subpopulations) who become targets of a stalking campaign. For example, it appears reasonable to think that the involvement of third parties may precipitate the decision to contact the police, but there is no specific information in this regard in the current template used for writing the reports. Fifth, our quantitative analysis of the police reports was conducted through the lens of the Italian version of the stalking questionnaire constructed by the Network for Surviving Stalking under Dr. Sheridan [33]. The reports might also be analysed qualitatively, e.g. with content analysis. This might allow a better understanding of the language used by the victims to describe their experience, highlighting differences, e.g., in how discomfort is expressed in the storytelling of men and women victims. Lastly, a comparison between victims of the stalking that did and victims that did not turn to the police was not made. Data about the latter is just non-existent in the Italian landscape, except maybe retroactively when graver offenses are committed. Luckily, however, this is comparatively rare and would therefore not provide appropriate grounds for comparison (and, as mentioned above, we were anyway not granted access to these dossiers). Further research should thus analyse in depth the characteristics of the actors involved (victim, stalker, different kinds of third parties involved) and those of the stalking campaign, to single out other possible differences between men and women victims who decide to contact or not to contact to the police. The findings could help understand what variables may explain the decision to turn to the police, also allowing improvements to social information campaigns.

## Conclusion

Despite these limitations, the study yielded a good deal of information. It revealed that men tend to let a stalking campaign last for a long time before reporting it to police. This may have consequences on the victims’ wellbeing, especially in the emotional sphere. To worsen the situation, the coping strategies generally adopted by men are not effective and may even hamper the work of the police officers who must intervene and investigate the case. It might then be useful to strengthen and improve the government’s efforts to promote the public awareness of the whole stalking phenomenon; sex/gender characterizations should be stripped off the social campaigns to change the public perception of stalking as exclusively involving women [46]. The biased representation of stalking in the media and in the social campaigns is also likely to further diminish the tendency for men to protect themselves and to add a sense of shame to an already intense emotional burden. A different approach might legitimize men to recognize themselves as victims of stalking as well as allow third parties like relatives, friends, and colleagues to recognize the signs of victimization in men [21,32]. A prompt intervention by police officers could provide more suitable interventions for men and their families. Also, the schema employed in the interviews with victims of stalking should be improved and made more flexible and more capable of including “semantic”, “lived” information. Finally, in the promotion of individual and social awareness of stalking, the different roles and effectiveness of the various strategies ought to be emphasized: e.g., to collect evidence and to seek help are a lot more useful than other strategies in decreasing individual discomfort and increasing collective security, and also allow police officers to devise more appropriate interventions.
